# QSAR and Classification Study on Prediction of Acute Oral Toxicity of *N*-Nitroso Compounds

**DOI:** 10.3390/ijms19103015

**Published:** 2018-10-03

**Authors:** Tengjiao Fan, Guohui Sun, Lijiao Zhao, Xin Cui, Rugang Zhong

**Affiliations:** Beijing Key Laboratory of Environmental & Viral Oncology, College of Life Science and Bioengineering, Beijing University of Technology, Beijing 100124, China; fantengjiao2014@emails.bjut.edu.cn (T.F.); sunguohui@bjut.edu.cn (G.S.); cuixin1201@bjut.edu.cn (X.C.); lifesci@bjut.edu.cn (R.Z.)

**Keywords:** *N*-nitroso compounds, acute oral toxicity, QSAR, classification, toxicity mechanism

## Abstract

To better understand the mechanism of in vivo toxicity of *N*-nitroso compounds (NNCs), the toxicity data of 80 NNCs related to their rat acute oral toxicity data (50% lethal dose concentration, LD_50_) were used to establish quantitative structure-activity relationship (QSAR) and classification models. Quantum chemistry methods calculated descriptors and Dragon descriptors were combined to describe the molecular information of all compounds. Genetic algorithm (GA) and multiple linear regression (MLR) analyses were combined to develop QSAR models. Fingerprints and machine learning methods were used to establish classification models. The quality and predictive performance of all established models were evaluated by internal and external validation techniques. The best GA-MLR-based QSAR model containing eight molecular descriptors was obtained with Q^2^_loo_ = 0.7533, R^2^ = 0.8071, Q^2^_ext_ = 0.7041 and R^2^_ext_ = 0.7195. The results derived from QSAR studies showed that the acute oral toxicity of NNCs mainly depends on three factors, namely, the polarizability, the ionization potential (IP) and the presence/absence and frequency of C–O bond. For classification studies, the best model was obtained using the MACCS keys fingerprint combined with artificial neural network (ANN) algorithm. The classification models suggested that several representative substructures, including nitrile, hetero N nonbasic, alkylchloride and amine-containing fragments are main contributors for the high toxicity of NNCs. Overall, the developed QSAR and classification models of the rat acute oral toxicity of NNCs showed satisfying predictive abilities. The results provide an insight into the understanding of the toxicity mechanism of NNCs in vivo, which might be used for a preliminary assessment of NNCs toxicity to mammals.

## 1. Introduction

*N*-nitroso compounds (NNCs) are an important class of potent toxicants that widely exist in the environment and diet [1]. The carcinogenicity, mutagenicity and toxicity of NNCs and their metabolites have been evaluated in various experiments [2,3,4]. Among the 300 NNCs that have been tested for their carcinogenic potential, more than 90% were proven to be carcinogenic in a wide variety of animal species [5,6]. Human exposure to NNCs occurs mainly through food, tobacco products, drugs, car interiors, and cosmetics [7]. However, NNCs may also be synthesized endogenously from precursors and nitrosating agents, mainly in the stomach, leading to the formation of potentially carcinogenic compounds [8,9,10,11]. Due to the potentially harmful effects of these compounds, it is necessary to study the mechanism of action of their biological effects, particularly the structure-activity relationship (SAR).

Quantitative structure-activity relationship (QSAR) and classification methods are ideal alternatives to biological experiments. Not only because of their higher efficiency and lower cost, but they can also provide rapid assessment of the potential impacts of chemicals on human health and the environment, including lethality or non-lethal adverse effects, as well as being able to predict biological or physicochemical properties [12,13]. Thus, the European Union (EU) published REACH (Registration, Evaluation, Authorization and Restriction of Chemicals) regulation for promoting their applications in various fields in 2006. The QSAR and classification models have been developed as feedback to different legislation around the world (e.g., EU REACH) as well as to assist in reducing animal testing and designing greener chemicals [14,15,16].

Generally, the acute toxicity of most chemicals is mainly induced by a narcotic mechanism of action, which has been long termed as “membrane perturbation”. Narcotic compounds certainly accumulate within biological membranes, thus, a number of effects at the membrane occur. If a compound can be identified as being unreactive or narcotic, its acute toxicity to a variety of species can be predicted accurately from the structure alone [17]. However, a number of compounds have specific toxic mechanisms of action (e.g., inhibition of specific enzymes or electrophilic/nucleophilic reaction) [17]. For example, NNCs can produce alkyldiazonium ions through metabolic activation by specific enzymes or spontaneous decomposition, followed by attacking bio-macromolecules (e.g., DNA and proteins) to exert their toxicity [4].

Previous studies have reported some predictive models of the carcinogenic potential of NNCs. Based on “di-region theory”, a quantitative pattern recognition method performed for structure-carcinogenic activity relationship of NNCs gave rise to 97% correct classification using 10 descriptors [18]. In addition, the results suggested that the bifunctional alkylation between α and β sites or α and γ sites of NNCs provided important roles in their carcinogenesis. The support vector machine (SVM) and linear discriminant analysis (LDA) were used to develop a classification model of carcinogenic properties of 148 NNCs with seven descriptors [19]. The obtained results confirmed the discriminative capacity of the calculated descriptors and the total accuracy of SVM (95.2%) is better than that of LDA (89.8%). Using a topological substructure molecular descriptors (TOPS-MODE) approach, Helguera et al. constructed several QSAR models for predicting the carcinogenic effects of NNCs through different routes of administration for male and female rats [20,21]. Yuan et al. developed an LDA method to predict the carcinogenicity and further understand the carcinogenic mechanism of NNCs in rats using a TOPS-MODE approach. The results indicated that a good classification (carcinogenic and noncarcinogenic) value of 90.1% was obtained with a dataset of 111 NNCs [7].

Although several SAR studies in the perspective of carcinogenicity of NNCs have been reported, to our knowledge, there are still no related studies on the relationship between molecular structure or properties and acute oral toxicity of NNCs. In the present work, a dataset consisting of acute oral toxicity (LD_50_) of 80 NNCs to rats was used to establish the QSAR and classification prediction models. The developed models were assessed using various statistical parameters and an external validation set. Based on the analysis of these developed models, some important information in connection with toxicity can be obtained, which may help us better understand the bio-transformation and toxic mechanism of NNCs in vivo. Moreover, these QSAR and classification models may provide a way to evaluate and predict the toxicity of many other untested NNCs, before they have adverse effects on both humans and the environment.

## 2. Results and Discussion

### 2.1. QSAR Models

#### 2.1.1. Model Validation

The initial number of descriptors of MLR (multiple linear regression) model developed based on Dragon and DFT (density functional theory) were 457 after removal of constant value and high inter-correlated descriptors. Then, the further screening was executed by GA (genetic algorithm) [22] coupled with the MLR procedure, followed by the generation of 100 models. All 80 NNCs were ranked according to the toxicity value (−logLD_50_), then one was selected as the test set every five compounds and the remaining 64 compounds were used as the training set. According to the rule-of-thumb [23,24], the ratio of the number of compounds in the training set over the number of variables (descriptors) should have a value of at least 5, which allows the flexibility to build models using up to 13 descriptors in the present study. After utilizing QUIK (Q Under Influence of K) module, 44 models remained without multicollinearity. For acceptable QSAR predictive models, they should satisfy the following conditions [24,25]: (i) Q^2^_loo_ > 0.5; (ii) R^2^_ext_ > 0.6; (iii) (R^2^_ext_ − R_0_^2^)/R^2^_ext_ < 0.1 and 0.85 ≤ *k* ≤ 1.15 or (R^2^_ext_ − R’_0_^2^)/R^2^_ext_ < 0.1 and 0.85 ≤ *k*’ ≤ 1.15; (iv) |R_0_^2^ − R’_0_^2^| < 0.3. R_0_^2^ and R’_0_^2^ are the mean coefficients of determination of experimental versus predicted values and predicted versus experimental values for regressions through the origin, respectively. *k* and *k*’ are the corresponding slopes of regression lines through the origin. Finally, eight models were selected by MCDM (Multi-Criteria Decision Making) (Figure S1 in the Supplementary Materials), which Q^2^_loo_ values ranged from 0.7214 to 0.7533 (R^2^ = 0.7786 to 0.8071), as listed in Table 1. Among these models, six descriptors were observed with higher frequency than other descriptors, namely, MATS6p, MATS4i, SpMin7_Bh(i), JGI4, B01[C-O] and F04[C-O]. The best QSAR model with eight descriptors for the prediction of acute oral toxicity of NNCs was shown in Equation (1). The actual values of selected descriptors in the best QSAR model were presented in Table S1 in the Supplementary Materials.

−logLD_50_ = 2.86 + 0.28nR06 − 0.55MATS6p − 1.29MATS4i − 12.07JGI4 − 2.06SpMin7_Bh(i) − 0.50B01[C-O] − 0.35F04[C-O] − 7.36*E*_HOMO_ (1)

N_tr_ = 65, Q^2^_loo_ = 0.7533, R^2^ = 0.8071, R^2^_adj_ = 0.7796, F = 29.2961, RMSE_tr_ = 0.2661, CCC_tr_ = 0.8933 N_test_ = 14, Q^2^_ext_ = 0.7041, R^2^_ext_ = 0.7195, RMSE_test_ = 0.2847, Q^2^_F1_ = 0.7041, Q^2^_F2_ = 0.7032, Q^2^_F3_ = 0.7794, CCC_test_ = 0.8062, (R^2^_ext_ − R_0_^2^)/R^2^_ext_ = 0.0215, |R_0_^2^ − R’_0_^2^| = 0.2642.

**Figure 1:**
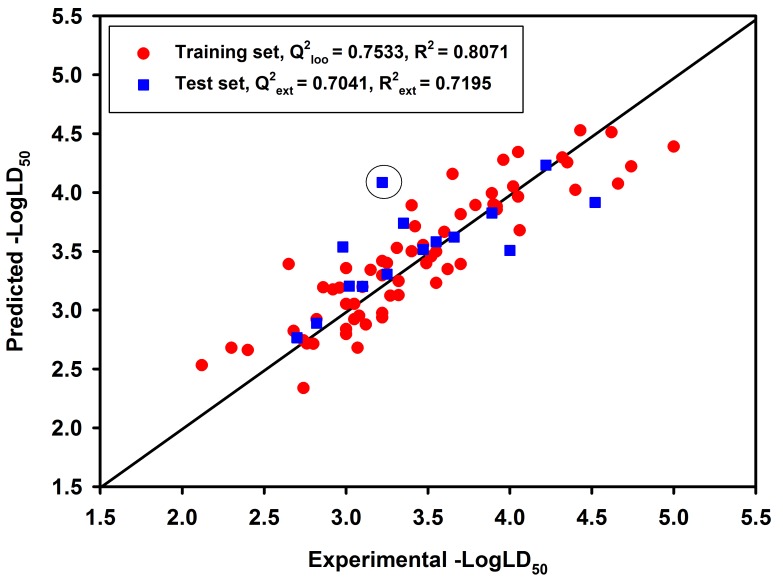
Experimental versus predicted toxicity values for compounds in the training set (red circle) and test set (blue square) of the best GA-MLR (genetic algorithm- multiple linear regression)-based quantitative structure-activity relationship (QSAR) model.

N_tr_ and N_test_ represent the number of compounds in the training and test sets, respectively. One compound in the original test set was removed because it was a predictive outlier (grey open circle in Figure 1). The relatively high quality of fitting parameters (R^2^, R^2^_adj_ and RMSE) and internal cross-validation correlation coefficient (Q^2^_loo_) indicate that the model has good internal fitting ability and robustness. A test set containing 14 compounds independent from the training set was used for an external validation to confirm the predictive ability of the MLR model. As shown in Table 2, the predictive ability of this model is high, which is reflected by Q^2^_ext_, R^2^_ext_ and RMSE_test_ as 0.7041, 0.7195 and 0.2847, respectively. The good external prediction was also observed with high CCC_ext_ (Concordance Correlation Coefficient) value (0.8062). Furthermore, a Y-scrambling procedure gave significantly lower statistical parameters (R^2^_Yscr_ = 0.1247, Q^2^_Yscr_ = −0.1890) when compared to the original model, thus we considered that the proposed QSAR model was not obtained casually.

The linear correlation between the experimental and predicted values from the best GA-MLR-based QSAR model (No. 21) was shown in Figure 1, in which red circles and blue squares represent compounds in training set and test set, respectively. All the studied NNCs are distributed evenly on both sides of the optimal line, indicating the good predictive power of this model. In addition, we applied the best prediction model on several NNCs from the ZINC database and found potentially toxic compounds without tested toxicity on rats, the results are listed in Table S2 in the Supplementary Materials.

#### 2.1.2. Outlier Analysis of MLR Model

**Figure 2:**
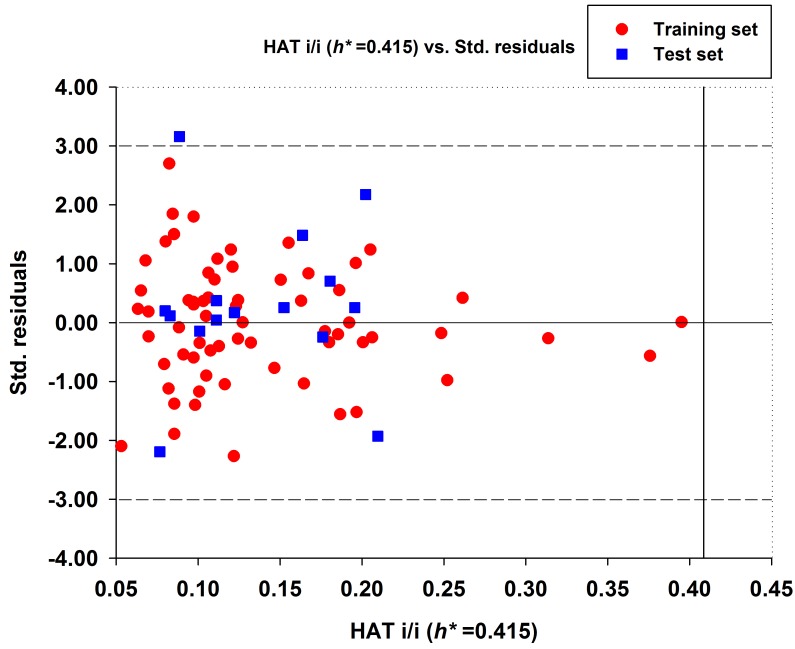
Williams plot for the best GA-MLR-based QSAR model. The transverse dash lines represent ±3 standard residual, vertical black line represents warning leverage h* = 0.415.

In developing the QSAR model, outliers strongly influence the regression parameters of the model. As a result, models should be re-established after outliers are removed. Williams plot, which represents the AD of the MLR model, is shown in Figure 2. It is very important to note that hat values of all compounds are lower than the critical hat value (h* = 0.415). Only one compound (**53**) in this study was identified as a predictive outlier because its standardized residual was slightly bigger than 3. In other words, the acute oral toxicity values of NNCs are generally well predicted by model 21 and they are reliable.

#### 2.1.3. Interpretation of Descriptors in MLR Model

**Figure 3:**
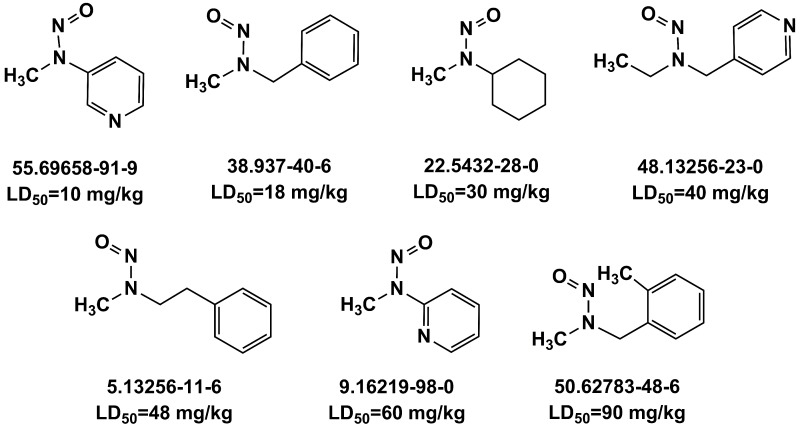
Several typical compounds that contain 6-membered rings in 22 high toxic *N*-nitroso compounds (NNCs).

Equation (1) indicates the best GA-MLR-based QSAR model consists of the following eight molecular descriptors: nR06, MATS6p, MATS4i, JGI4, SpMin7_Bh(i), B01[C-O], F04[C-O] and ***E*_HOMO_**. The corresponding types and chemical meanings of molecular descriptors are listed in Table 3, and the detailed explanation can be found in Handbook of Molecular Descriptors [26]. nR06 means the number of 6-membered rings, which is the only variable positively correlated with the high in vivo toxicity of NNCs. There were 7 compounds that contain 6-membered rings in the 22 high toxic NNCs, their molecular structures and LD_50_ values were shown in Figure 3. MATS6p is the Moran autocorrelation of lag 6 weighted by atomic polarizability, indicating a relationship between molecular polarizability and toxicity. According to the handbook of molecular descriptor [26], the Moran coefficient of the autocorrelation descriptors usually takes a value ranging from −1 to +1. Positive autocorrelation produces positive values of the coefficient whereas negative autocorrelation corresponds to negative values. MATS6p tends to have low values when the polarizabilities of bonded atoms are large. Matteo Cassotti et al. also found a relationship between molecular polarizability and acute aquatic toxicity of 546 organic molecules [27]. Polarizable molecules are usually considered as ‘soft’ species, which tend to react with other soft species, it thus appears that more-polarizable molecules tend to have higher toxicities, and this might be due to the formation of covalent bonds involving the HOMO and LUMO of soft acids and bases [27]. The MATS4i is also the Moran coefficient of the autocorrelation descriptors, while SpMin7_Bh(i) belongs to Burden eigenvalues. The MATS4i and SpMin7_Bh(i) are both related to ionization potential (IP), which is defined as the energy needed to extract one electron from a chemical system. The equation is shown as below:

IP = E(N_el_) − E (N_el_ − 1) (2)

where N_el_ is the number of electrons in the system. IP can be used to measure the capability of a molecule to give the corresponding positive ion. The low values of MATS4i correspond to the compounds that have C=C bonds. Zhang et al. [28] demonstrated a good relationship between epoxidation activation energies and IP, which means that the activation energy of epoxidation by P450s strongly depends on the conversion of the double bond in the olefin to a single bond in the product. There are also some NNCs containing olefins which can be activated by P450s to exert their toxicity to a certain degree. JGI4 is a kind of topological charge indices (Mean topological charge index of order 4) which can evaluate the charge transfer between pairs of atoms, and therefore the global charge transfer in the molecule [29,30]. B01[C-O] and F04[C-O] are both 2D atom pairs descriptors that describe pairs of atoms and bond types connecting them in 2D space. They represent the presence/absence of C–O bond and frequency of C–O bond at corresponding topological distance, respectively. There was a negative correlation between these two descriptors and in vivo toxicity of NNCs. The last descriptor ***E*_HOMO_** is a quantum chemistry descriptor. Molecules with high HOMO (highest occupied molecular orbital) energy values can donate their electrons more easily compared to molecules with low HOMO energy values, and hence are more reactive. Therefore, within the validity of the Koopman’s theorem, the ***E*_HOMO_** descriptor is also related to the IP, is a measure of the nucleophilicity of a molecule, and is important in modeling molecular properties and reactivity [31].

Considering all the molecular descriptors, acute oral toxicity of NNCs is mainly associated with three properties: Polarizability, IP, and the presence/absence and frequency of C–O bond. In addition, types of structural fragments (i.e., nR06) and charge/electrons transfer in molecules also affect molecular toxicity of NNCs.

### 2.2. Classification Models

#### 2.2.1. Data Set Analysis

In this study, a total of 80 NNCs collected from the US National Library of Medicine TOXNET ChemIDplus database were used for model building and validation. The 80 NNCs compounds were divided by the classification criterion of 200 mg/kg, then a dataset with 22 high toxic compounds and 58 low toxic compounds was obtained. The training set consisted of 15 high toxic and 41 low toxic compounds while the external test set contained 7 high toxic and 17 low toxic compounds. As an added precaution, it was verified that each set contained roughly the same percentage of high toxic compounds (training set = 26.8%, test set = 29.2%).

**Figure 4:**
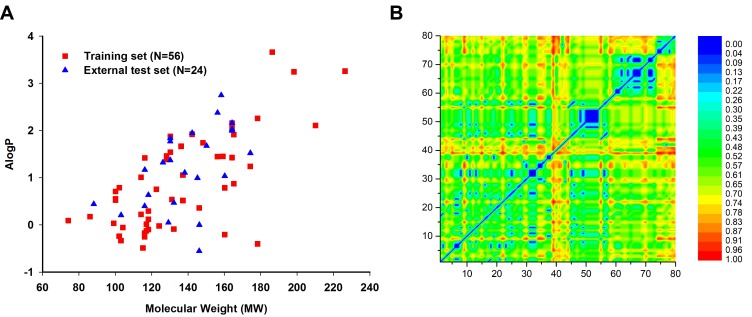
Chemical diversity analysis of the training and external test sets. (**A**) Chemical space was defined by molecular weight (MW) and Ghose−Crippen LogKow (ALogP). N represents the chemical number of different data sets. (**B**) Similarity heat map of Euclidian distance metrics calculated using MACCS keys fingerprint for the training and external test sets.

Chemical diversity is important to build a robust and reliable prediction model. We have investigated the chemical space distribution by calculating the molecule weight (MW) and Ghose−Crippen LogKow (ALogP) of the training set and the external test set [32]. The distribution scatter diagram was presented in Figure 4A. The scatter diagram showed that the chemical space of compounds in the external test set was within the scope of the training set. To further explore the chemical diversity of the data set, the Euclidian distance metrics of the data set was calculated. The training and external test sets were compared with each other, and the heat map of Euclidian distance metrics was shown in Figure 4B. It is clear that the similarity between the training and external test sets was low.

#### 2.2.2. Performances of 10-Fold Cross-Validation

**Figure 5:**
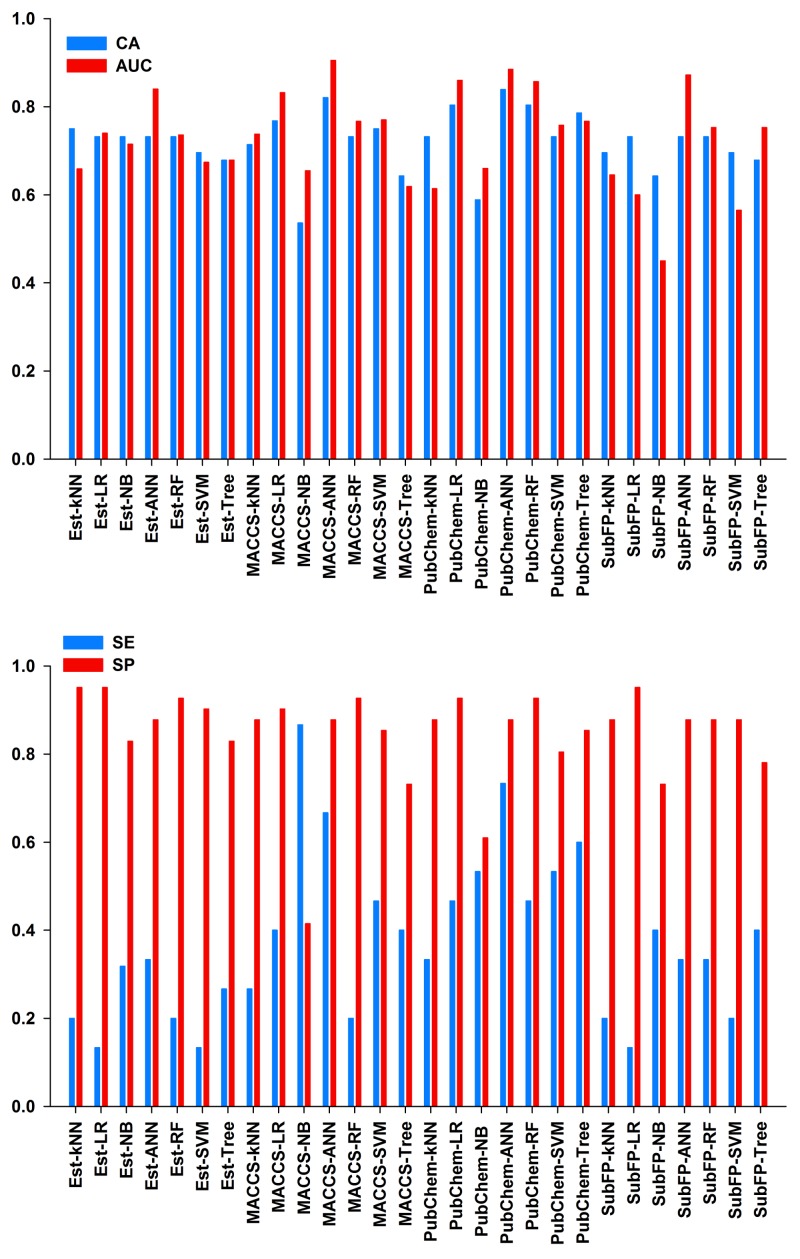
Performance of 10-fold cross-validation for the training set in 28 classification models. CA, classification accuracy; AUC, the area under the ROC curve; SE, sensitivity; SP, specificity.

In our classification study, we built the combinatorial predictive models by using four different fingerprints along with seven statistical algorithms. As a result, a total of 28 binary classification models were generated. The detailed evaluation results of these models are shown in Figure 5. The performance of these models was evaluated by 10-fold cross-validation, and the best models were selected based on the values of CA (classification accuracy) and AUC (the area under the ROC curve). As shown in Figure 5, most models had CA values more than 0.6, except for MACCS-NB and PubChem-NB models. Similarly, most models were obtained with AUC values higher than 0.6, except for the SubFP-NB and SubFP-SVM models. According to the results, the top eight ranking models were MACCS-ANN, PubChem-ANN, SubFP-ANN, PubChem-LR, PubChem-RF, Est-ANN, MACCS-LR and MACCS-SVM. Their CA values were 0.732–0.839 and AUC values were 0.770–0.905. The values of specificity (SP) were higher than that of sensitivity (SE) in all models, which means that all models have higher prediction accuracy for low toxic compounds rather than high toxic compounds. The underlying reason might be that more low toxic compounds existed in the data set. The detailed performances of the top eight models are shown in Table 4. By comparing the performance of four fingerprints, we could draw a conclusion that the MACCS and PubChem fingerprints are appropriate for the classification study of NNCs regarding in vivo toxicity. Based on the well-defined structural fragments dictionary, MACCS molecular fingerprint is full of structural information [33]. In previous studies, MACCS and PubChem fingerprints had also been proven to outperform other fingerprints in classifier models [33,34]. By contrast, the Est fingerprint performed worst when the same machine learning methods were used. This might due to the nature of the Est fingerprint, where only 79 bits signified substructure patterns are involved. It seems that the 79 bits are too short to represent diverse fragments of all compounds. When using the same molecular fingerprint, ANN and LR algorithms were better than other methods (*k*NN, NB, SVM, RF and Tree) in this study. For example, the 10-fold cross-validation results using MACCS showed that the AUC values of MACCS-ANN and MACCS-LR models were 0.905 and 0.832 respectively, whereas the values were 0.738, 0.655, 0.770, 0.767 and 0.619 in MACCS-*k*NN, MACCS-NB, MACCS-SVM, MACCS-RF and MACCS-Tree models, respectively.

#### 2.2.3. Performance of External Test Set

The external test set was utilized for testing the top eight models. The performance of the eight best models for test set is also shown in Table 4. The CA and AUC values ranged from 0.667 to 0.875 and 0.626 to 0.992 for external test set, respectively. Except for PubChem-RF model and the model using SubFP fingerprint, all models exhibited good predictive performance for external test set with both CA and AUC values higher than 0.7. Similar to the training set, the values of SP in these models were significantly higher than that of SE, which reflected almost perfect predictive ability for low toxic compounds in these models. Especially, the highest accuracy of 100% for low toxic compounds (SP) was obtained in MACCS-ANN, Est-ANN and MACCS-LR models. However, in all generated 28 models, only the MACCS-SVM model had good accuracy for high toxic compounds with SE value of 0.71. We supposed that the higher predictive accuracy for low toxic compounds in external test set was caused by the imbalance of high toxic compounds and low toxic compounds with a ratio of 0.292. Among these models, the MACCS-ANN model (CA = 0.792, AUC = 0.992) yielded the best performance, followed by MACCS-LR (CA = 0.875, AUC = 0.958) and MACCS-SVM (CA = 0.875, AUC = 0.899) models for the external test set. It is worth noting that the longer bits of fingerprint did not always get better results. For example, the PubChem fingerprint that contains 881 bits substructure patterns did not produce the best classification performance in this study. On the basis of our results in training and test sets, the MACCS fingerprint might be the best choice for the classification study of NNCs in terms of in vivo toxicity. Seven machine learning methods were used in this study. From the overall prediction performance, we can conclude that two algorithms, namely ANN and LR, produced the best results, in which models using ANN algorithm were slightly superior to those models using LR algorithm. As we know, LR is a widely used technique of choice for statistical modeling in which the outcome of interest is binary [35]. ANN is a type of algorithm that has great potential to execute nonlinear statistical modeling and provide a new alternative to LR, the most commonly used method for establishing predictive models for binary outcomes in medicine [35]. ANN offers a set of advantages, such as detecting complex nonlinear relationships between dependent and independent variables, detecting all possible interactions between predictor variables, requiring less formal statistical training and the availability of multiple training algorithms. We recommend that the outstanding performance of ANN in 10-fold cross-validation and external validation is because of its special algorithm [35]. In general, the prediction results showed the stable robustness and good prediction accuracy of the models.

#### 2.2.4. Identification of Privileged Substructures as Structural Alerts

To investigate the structural features between high toxic and low toxic NNCs, the IG method and substructure frequency analysis were performed to recognize privileged substructures (fragments) in the training and external test sets based on SubFP fingerprints [33,36,37]. The higher the information gain value, the more important the substructure. These chemical features contribute to investigate the relationship between structure and the acute oral toxicity of NNCs. Details of IG values and frequencies of each fragment occurred in the high and low toxic classes are shown in Table S3 in the Supplementary Materials. From the results of the IG analysis and frequency values of privileged substructures, we found 30 substructures responsible for in vivo toxicity of NNCs. Some representative privileged substructures and known compounds containing these substructures are listed in Table 5. Among these 30 substructures, the following five substructures, namely nitrile, Hetero N nonbasic, Heteroaromatic, Alkylchloride, and Tertiary aliph amine appeared more frequently in high toxic class rather than low toxic class of NNCs (Table 5). This implies that these six substructures can be considered as structural alerts for high toxic NNCs in vivo, and then can be used as the screening alert fragments to predict potential toxicity of new potential NNCs. For example, the compounds *N*-Nitrosomethylaminoacetonitrile (**14**) containing a nitrile fragment and 2-Chloro-*N*-methyl-*N*-nitrosoethanamine (**27**) containing a chloroethyl fragment are two highly toxic agents with LD_50_ values of 45 and 22 mg/kg, respectively. It has been mentioned in a previous study that nitrile was a potentially toxic fragment [38]. Nitrile compounds (e.g., acetonitrile, acrylonitrile, and propionitrile) can release the cyanide anions through hydrolysis to exert their high toxicity [38]. The cyanide anion could affect the central nervous system and the heart by inhibiting cytochrome c oxidase. Hetero N nonbasic can be defined as an aromatic nitrogen atom having two further total connections or an aromatic nitrogen atom affording a charge of +1 with three further total connections. While another opinion suggested that hetero N and heterocycle might be only the background noise of models, or they may be parts of some toxic substructures not defined in the fingerprint [39]. The alkylchlorides are potentially alkylating agents towards DNA. In compounds containing these fragments, the electron withdrawing effect of the Cl atom increases the electrophilic character of the carbon, followed by forming carbocations and resulting in DNA damage. For example, chloroethylnitrosoureas are an important type of anticancer agents, they exert anticancer activity through chloroethylating DNA guanine and ultimately produce G–C interstrand crosslinks [40,41,42,43]. Other toxic compounds containing alkylchlorides include nitrogen mustards, epichlorohydrin, dichloromethane, dichloroethane and so on. Tertiary aliph amine compounds usually undergo metabolic activation to generate a number of oxidative products including *N*-dealkylation, ring hydroxylation, α-carbonyl formation, *N*-oxygenation, and ring opening metabolites through the formation of iminium ion intermediates [44]. Some environmental pollutants and therapeutic pharmaceuticals and their related metabolites containing a tertiary amine structure have the potential to form iminium intermediates that are reactive toward nucleophilic macromolecules, including the piperazines, piperidines and related compounds, pyrrolidines and *N*-alkyltetrahydroquinolines [44]. The substructure fragments were also analyzed by the MoSS module in KNIME [45]. The results indicated that 41 fragments were obtained for acute oral toxicity of NNCs. The detailed results are listed in Table S4 in the Supplementary Materials. Pyridine (Hetero N nonbasic) and nitrile derivatives have a larger proportion in Moss results, which is consistent with the IG results. The unique substructure characteristics detected by MoSS are imine and hydrazine fragments. Imine derivatives (Schiff base) are unstable and undergo hydrolysis to give the corresponding amine and carbonyl compounds, in which the latter (e.g., aldehydes or ketones) contain potential carbocations which act as electrophiles to form adducts with DNA. Compounds containing hydrazine fragments can be activated by endogenous substances such as metal ions or enzymes (e.g., cytochrome P450-dependent oxidases and flavin monooxygenases) to form carbocations and carbon-centered radicals, resulting in reactive radical species that cause DNA damage [33].

## 3. Materials and Methods

### 3.1. QSAR Study

#### 3.1.1. Data Preparation

The in vivo toxicity data of 80 NNCs were carefully collected from the US National Library of Medicine TOXNET ChemIDplus database in terms of 50% lethal dose concentration (LD_50_) [46]. We selected oral LD_50_ values in rats as the endpoint in this study, since most of experiments chose the oral route to estimate the toxicity [2,47]. Compounds that contain at least 1 *N*-nitroso group substituent were collected from the database. To date, this is the largest dataset that contains rodent toxicity data for NNCs as far as we know. Most regression algorithms depend on normally distributed data, so if the data are not normally distributed, a numerical transformation should be performed to obtain a normal distribution. In this study, all the original LD_50_ values were converted into the corresponding −logLD_50_ values and were used as the dependent variables in QSAR analysis. The –LogLD_50_ values for the dataset range from 2.12 to 5.00, suggesting the data are adequately distributed for QSAR study. The name, CAS no. and toxicity values of NNCs are listed in Table 6.

#### 3.1.2. Calculation of Descriptors

Quantum chemistry calculations were prevalently used in the study of QSAR modeling [48,49,50]. The density functional theory (DFT) level of approximation for chemistry is suitable for many applications because of the better accuracy and the relative computational efficiency [51,52,53]. In the present study, before calculating molecular descriptors, all chemical structures of NNCs were generated by using the Gaussview 5.0 software (Gaussian, Inc., Pittsburgh, PA, USA), and then were optimized by DFT method using the Gaussian 09 program [54] at the B3LYP functional (the standard Becke’s three-parameter exchange potential and the Lee-Yang-Parr correlation functional, Gaussian, Inc., Wallingford, CT, USA) and 6-311++G(d,p) basis set. Frequency analyses on the optimized geometries ensure the geometry is an accurate saddle point rather than a transition state. A set of quantum chemical descriptors were calculated after the geometry optimization, such as dipole moment (*μ*), total energy (*E)*, the highest occupied molecular orbital energy (*E*_HOMO_), the lowest unoccupied molecular orbital energy (*E*_LUMO_), *E*_LUMO_ − *E*_HOMO_ gap, the bond lengths (*B*) and the bond angles (*A*). The DRAGON [55] software (version 7.0) was used to obtain the 0-2D (two-dimension) molecular descriptors. As most 3D descriptor groups encoding 3D structures were found to be sensitive to the quantum chemical calculation method [56] which can influence the accuracy of QSAR model, we therefore excluded the 3D descriptors. The total number of 0-2D descriptors was 3822. Finally, the quantum chemistry descriptors were combined with the 0-2D descriptors generated by DRAGON software to establish the QSAR models. The wide range of descriptors will facilitate the finding of hidden important variables.

#### 3.1.3. QSAR Modeling and Model Evaluation

QSARINS 2.2.2 software (Varese, Italy) [57,58] was used to develop QSAR models by means of GA and MLR methods. After all types of molecular descriptors were generated, we performed the pre-filtration prior to modeling. The constant or near-constant values (>80%) and the highly inter-correlated descriptors (>95%) were eliminated due to statistical insignificance. All the compounds were ranked according to the toxicity value (−logLD_50_), then one was selected as the test set every five compounds, and the remaining compounds were used as the training set. A training set was used for constructing QSAR models, whereas a test set was used for evaluating the external predictive ability of the models. All subsets and GA tools of QSARINS 2.2.2 software were utilized for descriptor selection. First, all low-dimensional models (up to 2–3 descriptors) were calculated using the all subset facility to gain an insight into the best descriptors encoding the effect and to avoid a completely random start of the GA. The core of chromosomes of the initial population for the GA was the best subset of descriptors determined at this step. Then, GA was utilized to detect the solution space by maximizing the leave-one-out (LOO) cross-validation correlation coefficient (Q^2^_loo_) as the fitness function. To obtain the best variables, the population size, mutation rate and number of generations were set as 200, 20 and 2000, respectively [23,56]. Q^2^_loo_ was chosen as it provides a measurement of model stability and robustness. Following this procedure repeatedly, a population of good models was generated.

The statistical quality and internal predictive ability of QSAR models were evaluated using the coefficient of determination R^2^ and modified form R^2^_adj_, root mean square error (RMSE) and Q^2^_loo_. The QUIK rule (Q Under Influence of K) [59] was used to test the inter-correlation among descriptors and was set to 0.05 to eliminate models with high multicollinearity. The external predictive ability of the models was assessed through the test set and evaluated by Q^2^_ext_, Q^2^_ext_ = 1 − PRESS/SD, where PRESS is the sum of squared deviations between the experimental values and the predicted value for each molecule in the test set, and SD is the sum of squared deviations between the experimental values of the test set molecules and the mean experimental value of the training set molecules [25]. Q^2^_F1_ [60], Q^2^_F2_ [61], Q^2^_F3_ [62,63], Concordance Correlation Coefficient (CCC) [64,65], CCC_ext_ [66,67] and RMSE_ext_ are also involved. A Y-scrambling procedure (2000 iterations to check the fitting of the randomly reordered Y-data) was also performed to evaluate the possibility of the chance correlation in the QSAR models. The dependent variables (−LogLD_50_) were randomly shuffled and new QSAR models were established using the original independent variable matrix. If the QSAR model obtained by shuffling the –LogLD_50_ values gave significantly lower coefficients of determination than the original model, we considered that the proposed QSAR model was not obtained casually. These parameters were calculated according to the following equations:

(3) $$R^{2}=\frac{\sum_{i=1}^{n}{\left(\hat{y_{i}}-\bar{y}\right)}^{2}}{\sum_{i=1}^{n}{\left(y_{i}-\bar{y}\right)}^{2}}\;$$

(4) $$\;R_{adj}^{2}=1-\frac{n-1}{n-k-1}\left(1-R^{2}\right)\;$$

(5) $$RMSE=\sqrt{\frac{\sum_{i=1}^{n}{\left({\hat{y}}_{i}-y_{i}\right)}^{2}}{n}}\;$$

(6) $$Q_{loo}^{2}=1-\frac{\sum_{i=1}^{n}{\left(y_{i}-{\hat{y}}_{i}\right)}^{2}}{\sum_{i=1}^{n}{\left(y_{i}-\bar{y}\right)}^{2}}\;$$

where *y_i_* and $\bar{y}$ are the actual and average activities and $\hat{y_{i}}$ are predictive activities.

The Multi-Criteria Decision Making (MCDM) method included in QSARINS 2.2.2 software was used to summarize the model performances relevant to internal and external validations as scores [56,57]. The scores range from 0 to 1, where 0 and 1 represent the worst and the best validation criteria, respectively. After numerous rounds of trials, models were finally selected with the best MCDM score, fulfilling the statistical thresholds for fitting, internal and external validation, and with the least possible number of descriptors [66,68].

#### 3.1.4. Application Domain

To consider the scope and limitations of the proposed models, the applicability domain (AD) was considered. In other words, the AD describes the range of chemical structures for which the models are considered to be applicable. The predicted values are reliable only for those compounds fall on the AD. The AD of each model was evaluated by the leverage approach [69]. Williams plot, which is a plot of standardized cross-validated residuals versus leverages (hat values, *h*), was used to visualize the outliers in both the structural and the response spaces. The critical hat value of structural threshold was set as h* = 3(*p* + 1)/*n*, where p is the number of descriptors of the model and n is the number of training compounds. If h > h*, a compound will be identified as an outlier. For the training set, compounds with h > h* seriously affect the statistical parameters of models, so they were removed, and the model was calibrated again. For the test set, if compounds are observed with h > h*, their predicted values were unreliable. A critical value of 3 for the standardized residual in response space is usually used to identify statistical outliers. Response outliers in MLR models were identified if its predicted value is higher than ±3 standardized residuals.

### 3.2. Classification Study

#### 3.2.1. Data Preparation

Before the classification study, we conducted a preliminary test to determinate the classification criterion. The same dataset of 80 NNCs used in QSAR studies was divided into three different levels of toxicity (50, 100, and 200 mg/kg, respectively). The results obtained from the preliminary test indicated that a toxic level with 200 mg/kg as the classification criterion had the best performance of classification. Finally, a dataset containing 22 compounds with high toxicity and 58 compounds with low toxicity was obtained. All these compounds were then randomly divided into a training set and a test set with a ratio of 7:3. A complete list of the compounds’ classification is presented in Table 6.

#### 3.2.2. Molecular Fingerprints

Molecular fingerprints are developed to describe chemical structures in a chemical database and widely used in similarity searching and classification. Therefore, substructure features in each fingerprint dictionary are defined to cover full of representative substructures. In this case, a molecule was described as a binary string of structural keys. SMiles Arbitrary Target Specification (SMARTS) is a language used for describing molecular patterns and properties using rules that are extensions of simplified molecular input line entry specification (SMILES) [70]. Different substructure patterns with SMARTS lists were predefined in a dictionary. For a SMARTS pattern, if a substructure existed in the given molecule, the corresponding bit was set to “1” and otherwise set to “0” [70]. Four fingerprints were used in our study, including the Estate fingerprint (Est, 79 bits), MACCS keys (166 bits), PubChem fingerprints (881 bits), and Substructure fingerprint (SubFP, 307 bits). All these four fingerprints were calculated by the PaDEL-Descriptor program [71].

#### 3.2.3. Machine Learning Methods

Seven machine learning methods were used to build the classification models. They are *k*-nearest neighbor (*k*NN), Logistic Regression (LR), Naïve Bayes (NB), Artificial Neural Network (ANN), Random Forest (RF), Support vector machine (SVM), and Tree. The seven methods were performed using Orange Canvas 3.11 software (freely available at https://orange.biolab.si/).

*k*-nearest neighbor (*k*NN): *k*NN is a nonparametric method to classify objects based on nearest training samples in the feature space. For each test sample Z = (x′, y′), the list of its nearest neighbor was determined by the algorithm calculated the distance or similarity between each training example (x, y) [72]. After that it can be classified on the basis of the majority of the nearest neighbors. In order to reduce the impact of k (the number of nearest neighbors) value, a distance-weighted method was utilized. In this study, we chose the Euclidean distance and distance-weighted parameters and the k value was set to 5.

Logistic Regression (LR): LR was developed by statistician David Cox in 1958 [73,74], which has usually been applied to a binary dependent variable. The two possible dependent variable values can be labeled as symbols of “0” and “1”, which represent results such as pass/fail, win/lose, alive/dead or yes/no, respectively.

Naïve Bayes (NB): The NB classifier method is a simple classification method based on the Bayes rule for the conditional probability [75]. This method allows users to categorize compounds in a data set based on the equal and independent contribution of their attributes. The prior probability can be directly estimated from the training set since it is the same to all of the classes, while the marginal probability is ignored. In this study, the default settings in Orange were applied to perform the NB classification.

Artificial Neural Network (ANN): ANN has become a prevalent method which can be used for identifying complex nonlinear relationship for classification and regression [76]. The network consisted of three layers containing one input layer, one hidden layer, and one output layer. The ANN method in Orange 3.11 is a multi-layer perceptron (MLP) algorithm with backpropagation. In this work, the number of neurons per hidden layer was set to 200, and the rectified linear unit function (ReLu) was chosen as activation function for the hidden layer.

Random Forest (RF): RF was developed by Breiman, which is an ensemble learning method for classification and regression [77]. The forest is assembled by trees. Each tree is developed from a bootstrap sample from the training set. The tree grows up to maximum size without pruning. When developing individual trees, an arbitrary subset of attributes is achieved (hence the term “Random”), from which the best attribute for the split is selected. The final model is based on the majority of individually developed trees in the forest. The number of trees in the forest was set to 20.

Support vector machine (SVM): SVM is a machine learning technique that separates the attribute space with a hyperplane, thus maximizing the margin between the instances of different classes or class values. It was first developed by Vapnik and co-workers in 1995, which is a kernel-based algorithm for binary data classification and regression [78]. Polynomial kernel, Gaussian radial basis function kernel (RBF) and sigmoid kernel are the generally used kernel functions. The penalty coefficient C and slack variable γ should be introduced to make a compromise between linear separability and maximal margin. In this study, the RBF kernel was chosen, and the parameters C and γ were tuned on the training set by 10-fold cross-validation. Orange embeds a popular implementation of SVM from the LIBSVM package [79]. The linear function was chosen, and the cost was set to 1.00.

Tree: Tree is a simple algorithm that splits the data into nodes by class purity. It is a precursor to RF. Tree in Orange is designed in-house and can handle both discrete and continuous datasets. It includes decision nodes, branches, and leaves. A decision tree inputs an object or situation described by a number of properties and outputs a yes/no decision. An instance is classified by beginning at the root node of the decision tree, testing the attribute specified by this node, followed by moving down to the tree branch according to the value of the attribute [80]. In the pre-pruning process, the minimal instance in leaves is 3, and stops splitting nodes with fewer instances than 5. Other parameters of tree were used with the default values in Orange.

#### 3.2.4. Performance Evaluation

The 10-fold cross-validation and test set were used to evaluate the performance of all the established models. For 10-fold cross-validation, the training set was further divided in to ten subsets, nine of which were chosen as training sets and one subset as a test set in each run. After ten runs, each subset was used as a test set and the entire dataset was predicted. All models were evaluated by counting the numbers of true positive (TP), true negative (TN), false positive (FP), and false negative (FN) compounds. Further, the classification accuracy (CA), sensitivity (SE), and specificity (SP) were also calculated by the following equations:

CA = (TP + TN)/(TP + TN + FP + FN)(7)

SE = TP/(TP + FN)(8)

SP = TN/(TN + FP)(9)

The CA is the total percentage of both high toxic and low toxic compounds that were correctly predicted. The SE is the predictive accuracy of the high toxic compounds and the SP means the predictive accuracy of low toxicity. Further, the receiver operating characteristic (ROC) curve where the TP rate (or sensitivity) against the FP rate (1-specificity) was plotted. The area under the ROC curve (AUC) was also calculated. The values of AUC range from 0.5 to 1.0 [81], where 1 indicates a perfect classifier, 0.5 means the classifier has no discriminative power.

#### 3.2.5. Analysis of Privileged Substructures

The information gain (IG) [70] and substructure fragment analysis [82,83] were used to identify the privileged substructure fragments and the structural alerts. If a substructure was more frequently presented in the class of compounds with high toxicity, this substructure could be regarded as a privileged substructure involved in chemical toxicity. The frequency of a fragment in high toxic compounds was defined as follows:

(10) $$\mathrm{Frequency}\;\mathrm{of}\;a\;\mathrm{fragment}=\frac{N_{fragment}^{H}\times N_{total}}{N_{fragment\_total}\times N_{H}}\;$$

where $N_{fragment}^{H}$ is the number of compounds containing the fragment in the class of high toxic compounds; $N_{total}$ is the total number of compounds; $N_{fragment\_total}^{H}$ is the total number of compounds containing the fragment; and $N_{H}$ is the number of high toxicity compounds.

In addition, the MoSS module in KNIME (available online: http://www.knime.org/) was also used to search for substructure fragments that are frequently presented in a set of molecules. In the MoSS module, the “minimum fragment size” and “minimum focus support in %” values are important for fragment search. In our study, the two values were finally set to 4 and 3, respectively.

## 4. Conclusions

In this study, we developed the QSAR and classification models of a large set of 80 NNCs with their rat acute oral toxicity. All QSAR models were established by GA-MLR methods. A reasonable correlation (Q^2^_loo_ = 0.7533, R^2^ = 0.8071, Q^2^_ext_ = 0.7041, R^2^_ext_ = 0.7195) was obtained between experimental and predicted toxicity values for the NNCs studied in the best QSAR model with eight molecular descriptors. The robustness and fitting goodness of QSAR models were evaluated using LOO cross-validation, while the test set was used to assess the external predictive power. The QUIK rule was used to eliminate models with high predictor collinearity. The possibility of chance correlation of the best model was checked by a Y-scrambling procedure. All the classification models were obtained by four molecular fingerprints (Est, MACCS, PubChem and SubFP) combined with seven machine learning methods (*k*NN, LR, NB, ANN, RF, SVM and Tree). All these models were examined by 10-fold cross-validation and external test sets to evaluate their internal and external predictive performance. The best classification model was the MACCS-ANN model with Q and AUC values of 0.821, 0.905 and 0.792, 0.992 for the training set and external test set, respectively. Analysis of privileged substructures performed by IG and frequency analysis methods can identify some substructures (fragments) as structural alerts for acute oral toxicity of NNCs. The substructures were further tested and verified by MoSS analysis. From the results of GA-MLR-based QSAR and classification models, we can conclude that the polarizability, IP, the presence/absence and frequency of C-O bond, Nitrile, Hetero N nonbasic, Alkylchloride, Tertiary aliph amine can be regarded as main attributes for assessing in vivo toxicity of NNCs. We believe that the models we developed reflect major contributions to our knowledge of the toxicity of NNCs. Compared with GA-MLR-based QSAR models, the semi-quantitative classification models could determine toxic severity of compounds with high accuracy directly. All the proposed models can provide useful insights into the structural features responsible for the acute oral toxicity of NNCs and therefore could help to improve our understanding of the toxicity mechanisms in vivo for this class of compounds. In summary, our study not only provides useful tools for predicting the in vivo toxicity of NNCs quantitatively or semi-quantitatively, but is also helpful to estimate acute toxicity in assessment of environmental safety.

## Figures and Tables

**Table 1:** Fitting and internal validation parameters of GA-MLR-based QSAR models selected by Multi-Criteria Decision Making (MCDM).

No.	Model No.	Number of Descriptors	Descriptors	R^2^	R^2^_adj_	RMSE_tr_	CCC_tr_	F	Q^2^_loo_	RMSE_cv_	CCC_cv_	Q^2^_lmo_	R^2^_Yscr_	Q^2^_Yscr_
1	21	8	nR06 MATS6p MATS4i JGI4 SpMin7_Bh(i) B01[C-O] F04[C-O] HOMO	0.8071	0.7796	0.2661	0.8933	29.2961	0.7533	0.3010	0.8651	0.7379	0.1247	−0.1890
2	27	8	nR06 MATS6p MATS4i JGI4 SpMin7_Bh(i) P_VSA_MR_1 B01[C-O] F04[C-O]	0.8033	0.7752	0.2688	0.8909	28.5870	0.7432	0.3071	0.8596	0.7267	0.1247	−0.1856
3	29	8	D/Dtr06 MATS6p MATS4i JGI4 SpMin7_Bh(i) B01[C-O] F04[C-O] HOMO	0.8023	0.7740	0.2695	0.8903	28.3984	0.7504	0.3028	0.8632	0.7335	0.1268	−0.1880
4	33	7	MATS6p MATS4i GATS1m JGI4 SpMin7_Bh(i) B01[C-O] F04[C-O]	0.7872	0.7611	0.2796	0.8809	30.1220	0.7322	0.3136	0.8520	0.7169	0.1097	−0.1644
5	34	7	Mp MATS6p MATS4i JGI4 SpMin7_Bh(i) B01[C-O] F04[C-O]	0.7848	0.7584	0.2811	0.8794	29.6947	0.7262	0.3171	0.8484	0.7076	0.1100	−0.1636
6	36	7	MATS6p MATS4i JGI4 SpMin7_Bh(i) H-046 B01[C-O] F04[C-O]	0.7807	0.7538	0.2838	0.8768	28.9864	0.7276	0.3163	0.8491	0.7140	0.1077	−0.1700
7	37	7	ZM1Mad MATS6p MATS4i GGI4 SpMin7_Bh(i) B01[C-O] F04[C-O]	0.7797	0.7527	0.2844	0.8762	28.8222	0.7214	0.3199	0.8446	0.7045	0.1094	−0.1669
8	38	7	MATS6p MATS4i JGI4 SpMin5_Bh(s) P_VSA_MR_1 B01[C-O] F04[C-O]	0.7786	0.7514	0.2851	0.8755	28.6378	0.7223	0.3194	0.8463	0.7040	0.1104	−0.1621

**Table 2:** External validation parameters of GA-MLR-based QSAR models selected by MCDM.

No.	Model No.	Number of Descriptors	Descriptors	R^2^_ext_	RMSE_ext_	Q^2^_F1_	Q^2^_F2_	Q^2^_F3_	CCC_ext_	*k*	*k’*
1	21	8	nR06 MATS6p MATS4i JGI4 SpMin7_Bh(i) B01[C-O] F04[C-O] HOMO	0.5401 (0.7195)	0.3544 (0.2847)	0.5147 (0.7041)	0.5144 (0.7032)	0.6581 (0.7794)	0.7023 (0.8062)	0.9774 (0.9957)	1.0132 (0.9977)
2	27	8	nR06 MATS6p MATS4i JGI4 SpMin7_Bh(i) P_VSA_MR_1 B01[C-O] F04[C-O]	0.5100 (0.6534)	0.3709 (0.3080)	0.4685 (0.6538)	0.4681 (0.6527)	0.6255 (0.7418)	0.7003 (0.7934)	0.9784 (0.9944)	1.0110 (0.9994)
3	29	8	D/Dtr06 MATS6p MATS4i JGI4 SpMin7_Bh(i) B01[C-O] F04[C-O] HOMO	0.5153 (0.7175)	0.3659 (0.2908)	0.4862 (0.6912)	0.4823 (0.6902)	0.6355 (0.7697)	0.6767 (0.7914)	0.9742 (0.9933)	1.0159 (0.9999)
4	33	7	MATS6p MATS4i GATS1m JGI4 SpMin7_Bh(i) B01[C-O] F04[C-O]	0.4632 (0.5963)	0.3806 (0.3354)	0.4381 (0.5894)	0.4399 (0.5881)	0.6056 (0.6938)	0.6398 (0.7221)	0.9787 (0.9946)	1.0101 (0.9962)
5	34	7	Mp MATS6p MATS4i JGI4 SpMin7_Bh(i) B01[C-O] F04[C-O]	0.4712 (0.6587)	0.3813 (0.3116)	0.4381 (0.6455)	0.4377 (0.6443)	0.6041 (0.7356)	0.6508 (0.7622)	0.9752 (0.9944)	1.0138 (0.9977)
6	36	7	MATS6p MATS4i JGI4 SpMin7_Bh(i) H-046 B01[C-O] F04[C-O]	0.4726	0.3780	0.4478	0.4474	0.6109	0.6609	0.9804	1.0084
7	37	7	ZM1Mad MATS6p MATS4i GGI4 SpMin7_Bh(i) B01[C-O] F04[C-O]	0.6322	0.3295	0.5805	0.5802	0.7044	0.7851	0.9751	1.0171
8	38	7	MATS6p MATS4i JGI4 SpMin5_Bh(s) P_VSA_MR_1 B01[C-O] F04[C-O]	0.4710 (0.5055)	0.3786 (0.3721)	0.4461 (0.4944)	0.4457 (0.4927)	0.6097 (0.6229)	0.6702 (0.6971)	0.9855 (0.9941)	1.0030 (0.9946)

**Table 3:** Type and chemical meaning of molecular descriptors in the best QSAR model.

Descriptor	Type	Chemical Meaning
nR06	Ring descriptors	Number of 6-membered rings
MATS6p	2D autocorrelations	Moran autocorrelation of lag 6 weighted by polarizability
MATS4i	2D autocorrelations	Moran autocorrelation of lag 4 weighted by ionization potential
JGI4	2D autocorrelations	Mean topological charge index of order 4
SpMin7_Bh(i)	Burden eigenvalues	Smallest eigenvalue n. 7 of Burden matrix weighted by ionization potential
B01[C-O]	2D Atom Pairs	Presence/absence of C–O at topological distance 1
F04[C-O]	2D Atom Pairs	Frequency of C–O at topological distance 4
*E*_HOMO_	QM descriptors	Highest occupied molecular orbital energy

**Table 4:** Performance of the top eight models for training set and external test set in classification study ^1^.

Data Set	Model	CA	SE	SP	AUC	TP	TN	FP	FN
Training set	MACCS-ANN	0.821	0.67	0.88	0.905	10	36	5	5
	PubChem-ANN	0.839	0.73	0.88	0.885	11	36	5	4
	SubFP-ANN	0.732	0.33	0.88	0.872	5	36	5	10
	PubChem-LR	0.804	0.47	0.93	0.860	7	38	3	8
	PubChem-RF	0.804	0.47	0.93	0.857	7	38	3	8
	Est-ANN	0.732	0.33	0.88	0.840	5	36	5	10
	MACCS-LR	0.768	0.40	0.90	0.832	6	37	4	9
	MACCS-SVM	0.750	0.47	0.85	0.770	7	35	6	8
Test set	MACCS-ANN	0.792	0.29	1.00	0.992	2	17	0	5
	PubChem-ANN	0.708	0.29	0.88	0.765	2	15	2	5
	SubFP-ANN	0.667	0.29	0.82	0.626	2	14	3	5
	PubChem-LR	0.792	0.43	0.94	0.889	3	16	1	4
	PubChem-RF	0.708	0.14	0.94	0.693	1	16	1	6
	Est-ANN	0.750	0.14	1.00	0.790	1	17	0	6
	MACCS-LR	0.875	0.57	1.00	0.899	4	17	0	3
	MACCS-SVM	0.875	0.71	0.94	0.958	5	16	1	2

^1^ Notes: CA, classification accuracy; SE, sensitivity; SP, specificity; AUC, the area under the ROC curve; TP, the number of true positive compounds; TN, the number of true negative compounds; FP, the number of false positive compounds; FN, the number of true negative compounds.

**Table 5:** Privileged substructures in compounds with high toxicity identified by information gain and frequency analysis method.

No.	Description	SMARTS	General Structures	Representative Compounds	IG	F_H_
SubFP133	Nitrile	[NX1]#[CX2]	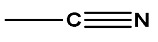	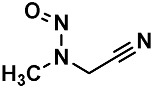	0.048	3.64
SubFP181	Hetero N nonbasic	[nX2,nX3+]	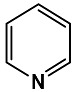	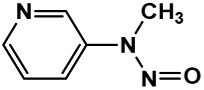	0.037	2.73
SubFP184	Heteroaromatic	[a;!c]	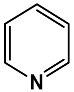	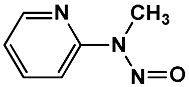	0.037	2.73
SubFP8	Alkylchloride	[ClX1][CX4]	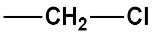	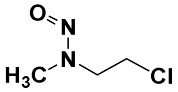	0.024	3.64
SubFP26	Tertiary aliph amine	[NX3H0+0,NX4H1+;!$([N][!C]);!$([N]*~[#7,#8,#15,#16])]	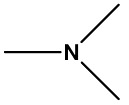	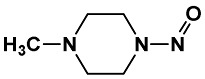	0.024	3.64

**Table 6:** Names, CAS no. and corresponding toxicity values of *N*-nitroso compounds used in this study.

No.	Name	CAS No.	LD_50_ mg/kg	Log (LD_50_)^−1^	Predicted log(LD_50_)^−1^
**1**	Diallylnitrosamine ^a^	16338-97-9	800 (L) ^b^	3.10	3.20
**2**	Dipentylnitrosamine	13256-06-9	1750 (L)	2.76	2.72
**3**	*N*-Methyl-*N*,4-dinitrosoaniline	99-80-9	1370 (L)	2.86	3.20
**4**	Nitroso-*N*-methyl-*N*-(2-phenyl) ethylamine	13256-11-6	48 (H) ^b^	4.32	4.30
**5**	*N*-Nitroso(2,2,2-trifluoroethyl)ethylamine ^a^	82018-90-4	960 (L)	3.02	3.20
**6**	Nitrosodibutylamine	924-16-3	1200 (L) ^b^	2.92	3.17
**7**	*N*-Nitrosodipropylamine	621-64-7	480 (L) ^b^	3.32	3.25
**8**	Nitrosoethylmethylamine	10595-95-6	90 (H) ^b^	4.05	4.34
**9**	2-Nitrosomethylaminopyridine ^a^	16219-98-0	60 (H)	4.22	4.23
**10**	Nitrosomethylaniline	614-00-6	225 (L)	3.65	4.15
**11**	Diisopropylnitrosamine	601-77-4	850 (L)	3.07	2.68
**12**	*N*-Nitrosobis(2,2,2-trifluoro ethyl)amine	625-89-8	300 (L)	3.52	3.46
**13**	*N*-Ethyl-*N*-*tert*-butylnitrosamine	3398-69-4	1600 (L) ^b^	2.80	2.71
**14**	*N*-Nitrosomethylaminoacetonitrile	3684-97-7	45 (H)	4.35	4.25
**15**	*N*-Butyl-*N*-(4-hydroxybutyl) nitro samine	3817-11-6	1800 (L) ^b^	2.74	2.34
**16**	*N*-Nitrosomethylvinylamine	4549-40-0	24 (H)	4.62	4.51
**17**	*N*-Nitroso-*N*-methylallylamine	4549-43-3	340 (L)	3.47	3.55
**18**	*N*-Ethyl-*N*-butylnitrosamine	4549-44-4	380 (L) ^b^	3.42	3.71
**19**	*N*-Nitrosodibenzylamine	5336-53-8	900 (L)	3.05	2.92
**20**	*N*-Nitroso-*N*-methylcyclohexylamine ^a^	5432-28-0	30 (H) ^b^	4.52	3.92
**21**	Nitrosomethyl-n-butylamine	7068-83-9	130 (H)	3.89	3.99
**22**	*N*-Ethyl-*N*-hydroxyethylnitrosamine	13147-25-6	7500 (L)	2.12	2.53
**23**	*N*-Amyl-*N*-methylnitrosamine	13256-07-0	120 (H)	3.92	3.85
**24**	Dinitrosodimethylethylenediamine	13256-12-7	125 (H) ^b^	3.90	3.90
**25**	Vinylethylnitrosamine	13256-13-8	88 (H)	4.06	3.68
**26**	*N*-Nitrososarcosine	13256-22-9	5000 (L)	2.30	2.68
**27**	2-Chloro-*N*-methyl-*N*-nitrosoethanamine	16339-16-5	22 (H)	4.66	4.10
**28**	*N*-Methyl(methoxymethyl)nitrosamine	39885-14-8	700 (L)	3.15	3.34
**29**	Methyl(acetoxymethyl)nitrosamine ^a^	56856-83-8	130 (H)	3.89	3.83
**30**	Acetoxymethylbutylnitrosamine ^a^	56986-36-8	1500 (L)	2.82	2.89
**31**	1-Methoxy-ethyl-ethylnitrosamine	61738-03-2	1000 (L) ^b^	3.00	2.84
**32**	Methoxymethyl-ethylnitrosamine	61738-04-3	540 (L)	3.27	3.12
**33**	1-Methoxy-ethyl-methylnitrosamine	61738-05-4	240 (L)	3.62	3.35
**34**	Acetoxymethylpropylnitrosamine	66017-91-2	1000 (L)	3.00	3.05
**35**	Methyl(butyroxymethyl)nitrosamine	67557-56-6	800 (L) ^b^	3.10	3.20
**36**	Acetoxymethyltrideuteromethylnitrosamine	67557-57-7	120 (H)	3.92	3.88
**37**	*N*-Nitroso-*N*-phenylhydroxylamine	148-97-0	490 (L) ^b^	3.31	3.53
**38**	*N*-methyl-n-benzylnitrosamine	937-40-6	18 (H) ^b^	4.74	4.22
**39**	4-(Methylnitrosoamino)benzaldehyde ^a^	7431-19-8	2000 (L)	2.70	2.76
**40**	3-(*N*-Nitrosomethylamino)sulfolan	13256-21-8	750 (L)	3.12	2.88
**41**	Aethyl-4-picolylnitrosamin	13256-23-0	40 (H)	4.40	4.02
**42**	*N*,*N*’-Dimethylnitrosourea	13256-32-1	280 (L)	3.55	3.50
**43**	*N*-Nitrososarcosine ethyl ester	13344-50-8	4000 (L)	2.40	2.65
**44**	4-Nitrosomethylaminopyridine	16219-99-1	200 (L)	3.70	3.81
**45**	*N*-Nitrosoethylisopropylamine	16339-04-1	1100 (L) ^b^	2.96	3.19
**46**	*N*-Nitrosotrimethylhydrazine	16339-14-3	95 (H) ^b^	4.02	4.05
**47**	*N*-Nitrosodiacetonitrile	16339-18-7	163 (H)	3.79	3.89
**48**	*N*-Nitroso-*N*-ethylbenzylamine	20689-96-7	250 (L) ^b^	3.60	3.66
**49**	*N*-Nitroso-*O*,*N*-diethylhydroxylamine	56235-95-1	1000 (L) ^b^	3.00	2.79
**50**	*N*-Nitroso-*N*-(2-methylbenzyl)methylamine	62783-48-6	90 (H)	4.05	3.96
**51**	*N*-Methyl-*N*-nitroso-(3-methylphenyl)methylamine	62783-49-7	600 (L)	3.22	3.41
**52**	*N*-Methyl-*N*-nitroso-(4-methylphenyl)methylamine	62783-50-0	400 (L) ^b^	3.40	3.89
**53 ^c^**	*N*-Nitroso-*N*-methyl-1(1-phenyl)-ethylamine ^a^	68690-89-1	600 (L)	3.22	4.00
**54**	*N*-Nitroso-*N*-methyl-2-(2-phenyl)-propylamine	68690-90-4	2100 (L)	2.68	2.82
**55**	3-Nitrosomethylaminopyridine	69658-91-9	10 (H)	5.00	4.40
**56**	*N*-Nitrosodiethylamine ^a^	55-18-5	220 (L)	3.66	3.62
**57**	*N*-Nitrosodimethylamine	62-75-9	37 (H)	4.43	4.53
**58**	*N*-Nitrosodiphenylamine	86-30-6	1825 (L)	2.74	2.74
**59**	*N*-Nitroso-3,6-dihydro-1,2-oxazine	3276-41-3	900 (L)	3.05	3.05
**60**	*R*(−)-*N*-Nitroso-2-methylpiperidine	14026-03-0	600 (L)	3.22	2.94
**61**	*S*(+)-*N*-Nitroso-2-methylpiperidine	36702-44-0	600 (L)	3.22	3.00
**62**	*N*-Nitrosoheptamethyleneimine ^a^	20917-49-1	283 (L)	3.55	3.58
**63**	*N*-Nitrosomorpholine	59-89-2	282 (L)	3.55	3.23
**64**	*N*-Nitrosopyrrolidine	930-55-2	900 (L)	3.05	3.35
**65**	1-Nitrosopiperazine	5632-47-3	2260 (L)	2.65	3.39
**66**	*N*-Nitrosopiperidine	100-75-4	200 (L)	3.70	3.39
**67**	*N*-Nitroso-tetrahydro-1,2-oxazine	40548-68-3	830 (L) ^b^	3.08	2.95
**68**	*N*-Nitrosoperhydroazepine ^a^	932-83-2	336 (L)	3.47	3.51
**69**	*N*-Nitrosoindoline	7633-57-0	320 (L)	3.49	3.40
**70**	*N*-Nitroso-*N*’-methylpiperazine ^a^	16339-07-4	100 (H) ^b^	4.00	3.51
**71**	*N*-Nitrosoazacyclononane	20917-50-4	566 (L) ^b^	3.25	3.40
**72**	3-Nitrosotetrahydro-1,3-oxazine	35627-29-3	600 (L)	3.22	3.29
**73**	*N*-Nitroso-1,3-oxazolidine	39884-52-1	1500 (L)	2.82	2.92
**74**	1-Amyl-1-nitrosourea ^a^	10589-74-9	560 (L)	3.25	3.30
**75**	*N*-Nitroso-*N*-butylurea	869-01-2	400 (L) ^b^	3.40	3.49
**76**	*N*-Nitroso-*N*-ethylurea	759-73-9	300 (L)	3.52	3.46
**77**	*N*-Nitroso-*N*-methylurea	684-93-5	110 (H)	3.96	4.27
**78**	Propylnitrosourea	816-57-9	480 (L)	3.32	3.13
**79**	*N*-Nitroso-*N*-methylbiuret ^a^	13860-69-0	450 (L) ^b^	3.35	3.73
**80**	Ethylnitrosobiuret ^a^	32976-88-8	1050 (L)	2.98	3.53

^a^ Test set in QSAR study; ^b^ Test set in classification study; ^c^ Outlier in the best GA-MLR-based QSAR model.

## Supplementary Materials

Supplementary materials can be found at http://www.mdpi.com/1422-0067/19/10/3015/s1.
