# Supplementary material for: QSAR and Classification Study on Prediction of Acute Oral Toxicity of N-Nitroso Compounds
Source: Int J Mol Sci. 2018 Oct 3;19(10):3015. doi: 10.3390/ijms19103015 (PMC6213880; doi:10.3390/ijms19103015)
Supplement: Supplementary file 1 [file ijms-19-03015-s001.zip › Supplementary materials-Figure S1.pdf]

Supplementary Materials: QSAR and Classification Study on Prediction of Acute Oral Toxicity of *N*-Nitroso Compounds

Tengjiao Fan, Guohui Sun, Lijiao Zhao, Xin Cui and Rugang Zhong

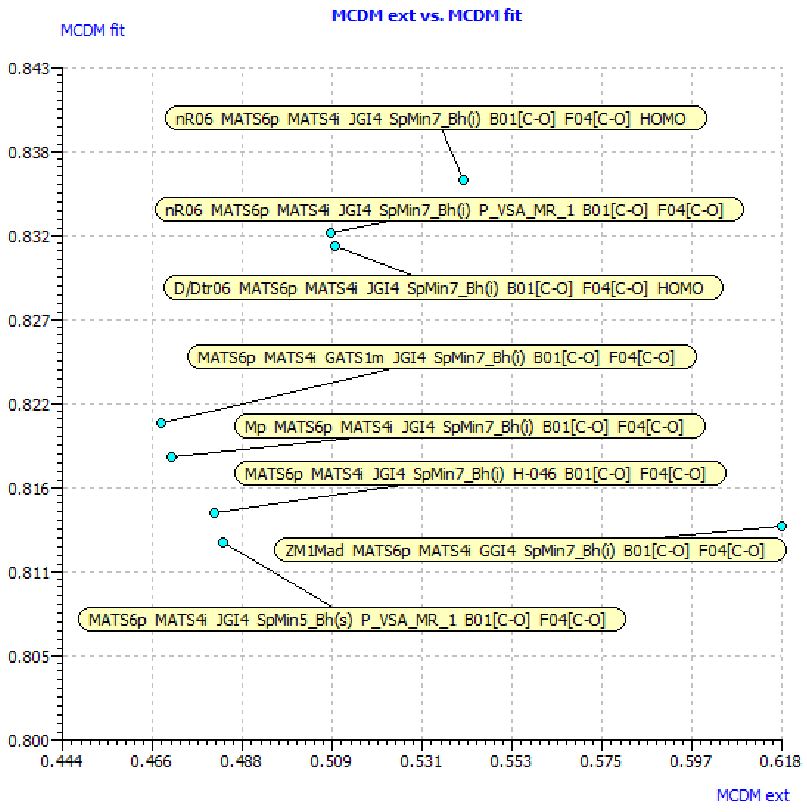

Figure S1. MCDM graph of the generated models.
